# A case report and literature review of cognitive malingering and psychopathology

**DOI:** 10.3389/fpsyt.2022.981475

**Published:** 2022-10-14

**Authors:** Tea Bosso, Flavio Vischia, Roberto Keller, Daniela Vai, Daniele Imperiale, Alessandro Vercelli

**Affiliations:** ^1^Department of Psychology, University of Turin, Turin, Italy; ^2^Cognitive Disorders Diagnosis and Treatment Centre, North-West Unit Amedeo di Savoia Hospital, ASL Città di Torino, Turin, Italy; ^3^Mental Health Department North-West Unit, Local Health Unit, ASL Città di Torino, Turin, Italy; ^4^Department of Neuroscience “Rita Levi Montalcini”, University of Turin, Turin, Italy

**Keywords:** cognitive malingering, psychopathology, neurocognitive disorder, neuropsychological assessment, performance validity tests (PVTs)

## Abstract

Malingering of cognitive difficulties constitutes a major issue in psychiatric forensic settings. Here, we present a selective literature review related to the topic of cognitive malingering, psychopathology and their possible connections. Furthermore, we report a single case study of a 60-year-old man with a long and ongoing judicial history who exhibits a suspicious multi-domain neurocognitive disorder with significant reduction of autonomy in daily living, alongside a longtime history of depressive symptoms. Building on this, we suggest the importance of evaluating malingering conditions through both psychiatric and neuropsychological assessment tools. More specifically, the use of Performance Validity Tests (PVTs)–commonly but not quite correctly considered as tests of “malingering”–alongside the collection of clinical history and the use of routine psychometric testing, seems to be crucial in order to detect discrepancies between self-reported patient's symptoms, embedded validity indicators and psychometric results.

## Introduction

Malingering is defined in DSM-5 (1) as “the intentional production of false or grossly exaggerated physical or psychological symptoms, motivated by external incentives such as avoiding military duty, avoiding work, obtaining financial compensation, evading criminal prosecution, or obtaining drugs” (1). This clinical phenomenon is probably present in 29% personal injury, 30% disability, 19% criminal, and 8% medical cases, with a prevalence ranging from 20 to 40% in forensic settings (2, 3). In the original malingering framework, the term Malingered Neurocognitive Dysfunction (MND) was defined as “the volitional exaggeration or fabrication of cognitive dysfunction for the purpose of obtaining substantial material gain or avoiding or escaping formal duty or responsibility” (p. 552) (4). Currently, the literature on this topic underlies the importance of self-reported somatic and psychiatric symptoms, in addition to malingering of mere cognitive dysfunction.

### Malingering: Detection vs. diagnosis

Suspicion of malingering sometimes occurs in clinical settings during the collection of patient's medical history, particularly when symptoms appear diagnostically incoherent or unlikely compared to more typical medical presentation, or if there is clear evidence of secondary gain (5). Overall, individuals usually undertake two main different strategies that compromise the validity of the examination: deliberately (i) reducing their physical, mental or cognitive capabilities, or (ii) overstating symptom complaints (6). Hence, for the purpose of *detection* of malingering, both strategies should be properly addressed through suitable instruments, namely: (i) Performance Validity Tests (PVTs) designed to detect performance that is below maximum functional capability (e.g., memory capacity), and ii) Symptom Validity Tests (SVTs) designed to detect over-reporting of symptoms (e.g., PTSD symptoms).

In considering the diagnosis of malingering, the clinician is explicitly making a determination of intent (6). For, whereas malingerers will engage in symptom over-reporting, not everyone who over-reports symptoms is a malingerer. In fact, the sequence of items and tests, symptom misinformation, inattentive responding, personality traits etc. can also lead to symptom over-reporting (7). In general, two medical conditions might be distinguished: factitious disorders and malingering (8). In the first condition (such as Munchhausen syndrome) the gain is psycho(patho)logical secondary benefit. In the second, instead, the motivation (gain) is external, such as receiving money. This distinction is not always clear-cut (9). Moreover, the two phenomena can sometimes overlap or co-occur (8). Finally, malingering “diagnosis” does not represent *ipso facto* the description of a disease. In fact, malingering is not a binary “all-or-none” phenomenon: an individual might be exaggerating actual difficulties by invention, perseveration, exaggeration or transference of symptoms (10).

For the diagnosis of malingering the use of DSM-5 and ICD-10 (11) criteria does not seem entirely satisfactory since it results in the accurate identification of only 13.6–20.1% of actual malingerers (true positives) (12). For this reason the American Academy of Clinical Neuropsychology (6) deemed Sherman, Slick and Iverson criteria (13)–which represent the updated version of Slick et al. criteria (4)- to be the most widely used model for detecting cognitive malingering within the empirical research on the subject (Table 1).

**Table 1:** The new multidimensional criteria for neurocognitive, somatic, and psychiatric malingering.

Criterion A	(A) Presence of an external incentive for feigning or exaggeration of deficits or symptoms at the time of examination
Criterion B	(B) Invalid presentation on examination indicative of feigning or exaggeration based on either (a) compelling inconsistencies indicative of deliberate exaggeration or feigning of deficits or symptoms during the evaluation, in both neurocognitive and psychiatric presentation (b) psychometric evidence of exaggeration or feigning of deficits or symptoms on performance validity tests (PVTs) or symptom validity tests (SVTs);
Criterion C	(C) Marked discrepancies between: test results, data/symptom report and the types of evidence: (1) Natural history and pathogenesis of the condition in question; (2) Records and other media; (3) Reliable collateral informant report
Criterion D	(D) Behaviors meeting Criterion B that cannot be fully accounted for by another developmental, medical, or psychiatric conditions

Modified from: Sherman et al. (13).

Finally, in order to overcome the limitations of DSM-5 model of malingering, it seems to be crucial to evaluate motivational patterns of patients who feign symptoms (14). At this respect, the literature on malingering suggests different explanatory models such as the *pathogenic model–*whereby malingering manifestation relies on the struggle between the unconscious illness and conscious production of symptoms-, the *criminological model–*whereby malingering is motivated by a desire to control and manipulate others-, and the *adaptational model* which explains motivations for malingering in terms of a risk–benefit analysis (15, 16). Crucially, these three models are not mutually exclusive.

### The assessment of cognitive malingering

The interest toward validity assessment, malingering, and effort has risen dramatically in neuropsychological research across the last 5 years (17). In the specific context of neuropsychological assessment, it is very important to consider both cognitive and functional difficulties and to cross-check multiple information resulting from clinical observation, psychometric tests and questionnaires in order to seize any discrepancy between level of performance at standardized tests and level of autonomy in everyday abilities. However, the simple strategies consisting in comparing cognitive test results (poor) and daily activities (preserved) might fail in cases of mild neurocognitive impairment, since this clinical condition is not usually accompanied by daily living impairments. Hence the importance of evaluating the validity of both performance and self-reports, as well as to subsequently include stand-alone cognitive PVTs (18, 19) embedded validity indicators (19, 20) and disorder-specific and/or general personality inventories during the assessment. In fact, individuals who malinger during a neuropsychological assessment typically exhibit a composite pattern of exaggerated cognitive, somatic and psychiatric symptoms (18, 21–24). Thus, all these symptomatic manifestations of malingering might be addressed during the evaluation. As suggested by literature (6), malingerers often show poor performance on measures of recognition memory, atypical error patterns on problem-solving tasks, poorer gross compared to fine motor testing, abnormally poor attention and Digit Span performances relative to memory and Vocabulary and poorer motor strength and speed, psycho-motor skills, attention and sensory findings relative to other neuropsychological functions. Although many cognitive tests and test batteries include purpose designed and/or actuarially derived embedded performance validity indices, it is considered best practice to utilize in addition PVTs (21, 25). In fact, PVTs are widely used in attempts to quantify effort and/or detect negative response bias during neuropsychological testing. Many PVTs ask participants to repeatedly make a *forced-choice* between two options–one of which is correct–so that random answering with no understanding of the task at hand should still produce a 50% accuracy rate. Due to their simplicity, most individuals–including those suffering from neurocognitive deficits–should do considerably better than that, and the worse an individual performs, the greater the probability of malingering. Numerous PVTs are available, including: Test of Memory Malingering (TOMM) (26), Word Memory Test (WMT) (27), Portland Digit Recognition Test (PDRT) (28), 21-Item Test (29), Coin-in-the-Hand Test (30) and Rey 15-Item Test (FIT) (31). Use of multiple performance validity tests (PVTs) may best identify invalid performance, though few studies in the Advanced Clinical Solutions (ACS) package have examined the utility and accuracy of combining PVTs. For instance, a recent study (32) showed that the word choice test (WCT) -generally useful for detecting invalid neuropsychological test performance- is less accurate among patients with severe memory impairment (AUC = 0.66; [AUC = area under the curve]) than among those with normal memory (AUC = 0.85) and mild memory impairment (AUC = 76). Another study (33) examined the Word Choice Test (WCT) in the Advanced Clinical Solutions (ACS) package to determine its utility alone and in concert with other two embedded measures: Reliable Digit Span (RDS), and Logical Memory Recognition (LMR). Bain and Soble (33) suggested that the ACS WCT has utility for detecting invalid performance in a clinical sample with likely cognitive impairment, though the embedded ACS measures (RDS and LMR) may have limited incremental utility, particularly in individuals with cognitive impairment. Finally, it has been demonstrated that classification accuracy statistics of WCT differs depending on different criterion grouping approaches, including: (1) failure of 2+ PVTs vs. failure of 0 PVTs, (2) failure of 2+ PVTs vs. failure of 0–1 PVT, and (3) failure of a stand-alone PVT vs. passing of a stand-alone PVT (Test of Memory Malingering). Therefore, criterion grouping approaches can impact PVT classification accuracy rates and resultant cutoff scores (34).

### Genuine psychopathology and malingering

Reports of impaired cognitive function are frequently present in many claims regarding psychiatric diagnoses (6, 35). Compared to patients effectively suffering from psychiatric disorders, malingerers try to produce an impression of illness or dysfunction that is believable and not obviously exaggerated, nonsensical or atypical by presenting exaggerated or simulated symptoms. Some of them succeed and receive the secondary gain while others fail in this endeavor and are detected because of the deviancy from typical symptom patterns of genuine cases (36, 37). However, when confronted with evidence of symptom exaggeration or fabrication, several diagnoses and/or explanatory constructs may need to be considered (Table 2). In addition, it is crucial to recognize that even in cases of well confirmed, genuine psychopathology, malingering may co-exist. Indeed, genuine psychopathology and symptom fabrication are not mutually exclusive (4, 26, 39, 40). Instead of considering this over-reporting as a pathognomic sign of malingering, multiple pathways to symptom over-reporting must be considered (7). Overall, cases in which evidence of malingering exists alongside the presence of clinically determined actual psychopathology or cognitive dysfunction are often especially challenging. As Morgan and Gervais asked: “how much of what I am seeing in the examinee is real (i.e., not exaggerated or feigned)?” (40).

**Table 2:** Suggested differential indicators of different clinical conditions in cases of exaggerated/fabricated neuropsychological dysfunction.

**Clinical condition**	**Required indicators**
Malingering	• Presence of a substantial external incentive; • Evidence from neuropsychological testing and/or self-report (not fully accounted for by psychiatric, neurological or developmental factors)
Malingering by proxy	Conclusive evidence that an examinee is acting under the influence of another person (directly observed) in position of influence or control (e.g., parent or spouse) when he feigned impairment
Secondary malingering	Feigning behaviors–otherwise, capable of meeting criteria for probable or definite malingering- are thought to wholly arise from legitimate cognitive/psychiatric dysfunction affecting self-control and/or the capacity to understand the moral/ethical nature or social/legal implications of feigning impairment would be given a diagnosis of secondary malingering. Secondary malingering can only result from a severe psychiatric (e.g., schizophrenia), neurological (e.g., severe traumatic brain injury), or neurodevelopmental disorder (intellectual disability), and cannot be caused by a mild neurological condition (e.g. mild traumatic brain injury)
Dissociative amnesia and Conversion disorder	The reported impairment is not volitional, but rather arises from intrapsychic conflicts and processes for primary gain.
Factitious disorder	The feigning of illness are not primarily directed toward obtaining psycho-social or material-legal secondary gains, but instead they provides gratification or relief from anxiety and intrapsychic conflict
Adjustment problem/disorder with specious symptoms (APSS/ADSS)	The feigning of symptoms is primarily directed toward (a) obtaining and maintaining psychological benefits (e.g., increased attention, affection, support from others); (b) managing problematic interpersonal relationships; and/or (c) escaping from adverse interpersonal situations or avoiding informal obligations (e.g., household chores or schoolwork)
Cogniform disorder	There is (a) a pattern of cognitive complaints or low scores on psychometric cognitive tests that cannot be fully explained by a neurological disorder, mental disorder, medical condition, effects of a psychoactive substance; (b) significant manifestations of cognitive dysfunction in widespread areas of everyday life
Neurocognitive hypochondriasis	• Fixed belief that to have neurologically-based cognitive impairment in the absence of any actual objective impairment • Hyper-vigilance to minor cognitive difficulties and failures, which are attributed to neurological injury or illness • No objective evidence of any impairment either real or feigned (i.e., test scores fall within the expected or normal range)
Oppositional-defiant presentations	• Refusal, nonsensical responses, and incompatible activities like looking away from or inappropriate use of test materials. • Presence of situational factors and/or be symptomatic of more pervasive or serious clinical conditions • No documented history of such problems

Modified from: Tracy (5); Slick and Sherman (38).

Among other psychiatric disorders, major depression represents a high-prevalence (7%) (1) mental disease with greater socioeconomic impact in terms of both direct (medications and hospitalization) and indirect (mortality, work absence and turnover, disability compensation) costs (41, 42). Exaggerated or feigned symptoms of depression are common in forensic examinations (2, 43). Recently, to detect them, validity scales such as the Malingered Depression Scale of the MMPI-2 (Md) (43) have been developed, with preliminary promising results. An alternative to MMPI-2 is represented by the Personality Assessment Inventory [PAI; (44)], and particularly the PAI NIM (Negative Impression) and the PAI-SOM (Somatic Complaints). Both the MMPI-2-RF and the PAI constitute solid SVTs with growing bodies of empirical support (45). Another important stand-alone symptom validity test is the 75-item, true/false Structured Inventory of Malingered Symptomatology (SIMS), which classifies feigned symptoms across five sub-scales: Psychosis, Neurological Impairment, Amnestic Disorders, Low Intelligence, and Affective Disorders. Despite, its lack of robust discriminant validity, several studies demonstrate convergent validity for the SIMS, as well as incremental validity when compared to clinical judgment based on interview and record review alone (46).

In general, when psychological disorders (e.g., depression) and ability deficits (e.g., memory) are claimed, appropriate assessment methods are required in order to evaluate response bias related to both and, consequently, to establish if psychiatric disorder *and* cognitive and/or functional decline are concurrently present (6). This said, when neuropsychological and psychological test results are unanimous in revealing multiple indicators of invalid test performance, even in cases of genuine psychopathology, it is most likely that exaggerated and or feigned/malingered psychopathology is present.

## Case description

DC was a right-handed 61 years-old male, with 8 years of schooling. When he came to our hospital, he was under house arrest, after his release from prison at the beginning of 2021, due to a sudden deterioration of physical and psychological conditions. During the interview, the patient was responsive but scarcely compliant and motivated to engage with the tasks that were presented to him, as well as seemingly disoriented to time, location and personal autobiographical information. His speech was normal, even though poor in form and content and characterized by confabulations and persecutory ideas. Comprehension seemed to be unfulfilled. His wife reported frequent episodes of spatial and temporal disorientation, together with attention and memory deficits, difficulties with logical reasoning and mental confusion. She also described a sudden loss of personal and instrumental autonomy in daily living over the last year. Finally, a variety of mood disorder symptoms has been described, such as: depressed mood, anxiety, apathy, psycho-motor slowdown, asthenia, lack of concentration and neurovegetative symptoms (with significant weight loss in the last year). Onset of essential tremor of the upper limbs had been reported since 2014, but neuroleptic treatment was not administered.

Different forensic reports from the prison period inconsistently feature diagnoses of either “mild cognitive impairment in vascular encephalopathy and anxious depressive disorders” or “major depressive disorder and major cognitive impairment with vascular origins.” Remarkably, none of the several MR and CT brain examinations carried out between 2018 and 2021 have revealed structural or functional evidence of brain damage. According to forensic documentation, the patient's judicial and medical history appear to be closely connected. The patient was first diagnosed with and prescribed medications for Major Depressive Disorder (DSM-IV) in 1993, the year in which his juridical problems first started. Since 1993 -concomitantly to his first legal persecution- psychiatric clinical records reported “episodes of somatization, functional disease, bulimia, emotional dysregulation and memory impairment.” For the last 10 years, the patient also reported problems related to attention and episodic memory, anomies, spatial disorientation and auditory hallucination. In 2016 and 2017, he was hospitalized with psycho-motor agitation, confusion and amnesia, and diagnosed with “dissociative amnesia.” Onset of seizure episodes of dubious nature, involving loss of consciousness, has been reported since 2016.

## Diagnostic assessment and results

Neuropsychological assessment included screening test (MMSE; ACE-R) and specific-domain tests for attention, memory, executive functions, language and visuospatial functions. Furthermore, a behavioral and functional examination was also conducted.

The analysis of the scores presented in Table 3 reveals an impaired performance across multiple domains, including: attention, memory, executive functions, language and visuospatial functions. In particular, DC exhibited comprehensive attentive difficulties with psycho-motor slowdown and a high number of intrusions during visual search tasks. He failed in counting from one to ten and in switching between numbers and letters. He also showed a severe global deficit in ability to consolidate and recall verbal and visuospatial information. Deficits of executive functioning were observed with difficulties in categorization, mental flexibility, ideomotor planning, reasoning, interference sensitivity, and inhibition. While DC's vocabulary appeared to be largely preserved in natural conversation, the formal assessment of basic language functioning revealed poor performances on graded naming tests and very poor phonologic and semantic fluency task performance. Visuospatial abilities seemed poorly preserved, with an inability to copy simple and complex figures, partly affected by an ambiguous tremor of the upper right limb. In addition to massive cognitive impairment, depressive- anxiety symptoms and significant loss of autonomy in daily living have been detected.

**Table 3:** Psychometric measures administered organized by domain of function.

**Domain of Function**	**Row score**	**Adjusted score**	**Equivalent score (ES)**	**Interpretative range**
**Screening**				
MMSE (Mini Mental State Examination Revised)	10/30	8.2	0	Impaired
ACE-R (Addenbrooke's Cognitive Examination)	32/100	37.5	0	Impaired
**Memory**				
Auditory Verbal Learning Test	1	0.75	0	Impaired
Corsi Block Tapping Test	1	1.15	0	Impaired
Digit span forward	2	2.13	0	Impaired
Prose Memory Test	2	1.75	0	Impaired
Rey-Osterrieth Complex Figure (Recall sub-test)	6.5	7	0	Impaired
**Attention**				
Attentive Matrices	28	24.75	0	Impaired
Trail Making test				
Part A Part B	Invalid Invalid	- -	0 0	Impaired Impaired
**Language**				
Aachener Aphasie Test- AAT (Denomination sub-test)	62	46	1	Low average
**Executive functions**				
Phonological Fluency	7	12.91	0	Impaired
Digit Span Backward	0	0.19	0	Impaired
**Visuospatial functions**				
Rey- Osterrieth Complex Figure (Copy sub-test)	14	15	0	Impaired
**Behavioral and functional assessment**				
ADL (Activity Daily Living)		1/6		Impaired
IADL (Instrumental Activity Daily Living)		0/5		Impaired
BDI-II (Beck's Depression Inventory)		21/63		Clinical relevance

Row scores have been converted into adjusted scores based on Italian norms based on age, sex, and education. Equivalent score ESs = 0 and 1 meaning “defective” and “borderline,” respectively; ES = 2 meaning “low-end normal”; ESs = 3 and 4 meaning “normal.” The test is considered invalid when the patient is not capable to perform the task for failure to understand and maintain instructions or for exceeding the maximum number of errors and/or the expected duration of the test.

The evidence of a multi-domain cognitive decline capable of interfering with DC's independence in everyday activities (e.g., cooking, bathing, managing medications and finances), seemed to suggest the diagnosis of “major neurocognitive disorder” (DSM-5). However, despite the presence of severe clinical manifestation, neuroimaging examinations showed no alteration on structural and functional connectivity. In particular, CT and MRI scan reported no signs of vascular brain injury, presence of clearly recognizable and symmetrical cerebral sulci, regularity in ventricular system, no mid-line shift. The absence of biomarkers from imaging, together with the presence of external incentives for malingering (i.e., avoiding imprisonment) and the existence of notable inconsistencies between medical and biographical history, clinical interview and patient's performance during neuropsychological assessment, forced us to proceed with the administration of screening measures specific for feigning, namely: Rey 15-Item Test (FIT) and Rey Dot Counting Test (DCT). In this respect, DC performed extremely poorly, obtaining the score of respectively 3/15 in FIT (cut off = 9) and E-score = 10 in DCT (cut off: E-score≥17), thus suggesting feigning or exaggeration of cognitive deficits during the previous assessment.

## Discussion

According to the Multidimensional Criteria for Neurocognitive, Somatic, and Psychiatric Malingering (13), DC seems to meet criterion (A) presence of an external incentive (i.e., DC's interest in keeping under house arrest and avoiding imprisonment); (B) compelling inconsistencies indicative of deliberate exaggeration or feigning of deficits or symptoms during the evaluation, and psychometric evidence of exaggeration or feigning of deficits or symptoms on PVTs (as previously discussed in case presentation); (C) marked discrepancies between obtained test data/symptom report and the types of evidence (i.e., natural history and pathogenesis of the condition in question); (D) Criterion B cannot be fully accounted for by another developmental, medical, or psychiatric condition. With regard to criterion D, however, the presence of a long-history depression, possibly exacerbated by lack of stimulation typical of any detention regime, has been corroborated, even if it has to be considered insufficient to preclude independence in basic activities of daily living). This result is consistent with the statement made by Sherman et al. (13) that malingering can co-occur in the presence of other psychiatric conditions (e.g., depression) (13). In this regard, DC was referred, in agreement with his wife, to the Mental Health Competence Center. Furthermore, a neuropsychological and psychiatric reassessment after 6 months was required, in order to evaluate the presence of any fluctuation in patient's performance and to consider any further use of assessment results in his clinical care and legal situation.

The case presented here appears interesting because of its implication for forensic psychiatry. Firstly, from a forensic psychiatric standpoint, a significant vagueness in the process of recollecting and interpreting symptoms together with a lack of pieces of evidence to support diagnosis is clear in the DC case presentation. In fact, DC's legal medical documentation seems to be dotted with different and inconsistent psychiatric and neurological diagnoses, not entirely supported by evidence from neuroimaging. In order to avoid or reduce this “diagnostic redundancy” it might be important not only to detect symptoms but, even more, to clinically contextualize them through a multidimensional psychiatric and psychological assessment based on collecting and combining different qualitative and quantitative information (8). Importantly, clinicians, while engaging the process of differential diagnosis, might be careful in considering and endorsing previous diagnosis of somatoform or functional neurological disorder, even more when many past medical diagnoses are listed in medical records (6). Clinicians are asked to keep in mind that somatic symptom disorder, conversion disorder, factitious disorder and malingering -despite being united by the inconsistency between subjective complaints and objective signs (47)- are though characterized by different levels of intentionality and they are aimed to different purposes. In addition to the above, it is important to remember that the co-occurrence of these different diseases might not be excluded regardless (4, 39, 40). In the case study presented here, for instance, a well-documented history of major depression appears to coexist with the fabrication or exaggeration of cognitive deficits, as revealed by malingering specific test administration. In this respect, it is important to firstly notice the difference between the tools available to psychiatry and neuropsychology in order to detect and diagnose, respectively, malingering of mental disorders and cognitive malingering. On the one hand, in detecting mental malingering, psychiatry has to face its inherent lack of reliable objective signs and its dependence on the subjective recollection and description of psychological phenomena, alongside the psychiatrist's expert knowledge and experience of mental disorder. Rogers et al. verify the application of 10 multiple and combined detection strategies for malingering of mental disorders, focused on two different markers: *excessive impairment* and *unexpected patterns* (12, 36, 37, 48). On the other hand, detecting cognitive malingering during mental state examination seems to be somewhat easier insofar as one can evaluate effort and look at probabilistic response patterns (5). In addition, the detection of impaired cognitive functions can be substantiated by neuroimaging (35, 49), which is also often capable of suggesting that the disease process is currently underway also in the absence of fully manifested clinical features (50). In any case it must be taken into account that the absence of abnormalities on neuroimaging does not rule out the possibility of legitimate cognitive deficits, nor does the presence of such abnormality automatically eliminate the possibility of exaggeration or fabrication of deficits and dysfunction. This is why PVTs and SVTs should be included in test batteries whenever examinees may be influenced by the possibility of substantial secondary gain, regardless of whether or not medical data (neuroimaging, GCS scores) is indicative of a brain injury.

## Conclusion

According to our review of the literature and the case study reported here, we suggest the importance of using proper tools from clinical psychiatry and neuropsychology (i.e., PVTs, SVTs, in addition to classical neuropsychological tests and psychological questionnaires), in order to correctly apply the label “malingering,” whenever necessary, without fear of legal consequences (51).

## Limitations of the study

During DC's neuropsychological assessment we used multiple measures of neurocognitive assessment listed in Table 3, but only two were specific to feigning, namely FIT and DCT, which are screening measures. We decided to use these two measures because they did not require specific training, in contrast to more defined measures of cognitive functioning such as the TOMM, WMT, VIP or WAIS-III digit span subtest. Furthermore, a survey of experts' practices (52) have found that 57% to 79% use at least one test designed specifically to assess for malingering when conducting an evaluation, with the two most commonly used tests for this purpose being the TOMM and the FIT. In this survey, one third of neuropsychologists reported that they “often” or “always” use the FIT when doing an evaluation, while about another one third indicated that they do use the test, but “rarely” (53). Overall, the Rey 15-Item Memory Test demonstrated to have high specificity across different population, but not equally satisfactory sensitivity (31). However, given the relative severity of consequences associated with a false positive (incorrectly labeling someone with genuine impairments as a malingerer) compared to a false negative (failing to detect a malingerer) in the legal system, specificity may be the important variable to examine here (54). Furthermore, although the FIT has a low sensitivity (49), it is frequently used because it is simple, inexpensive, and easy to score. With regard to DOT, using a cutoff of E-scores ≥17 resulted in 100% sensitivity in the forensic suspect group, 75% sensitivity in the civil litigation and disability suspect effort group, and a specificity of ≥90% for the clinical groups combined. These data demonstrate the ability of the DCT to detect non-credible cognitive symptoms in litigation/disability and forensic participants (54).

## Future directions

In the last 25 years, scientific research on malingering provided strong evidences supporting the effectiveness and accuracy of PVTs for detecting invalid neuropsychological test performance across medico-legal, clinical, and research settings. Nowadays, clinical neuropsychologists and psychiatrist have many different stand-alone and embedded measures at their disposal. However, the practice and science of performance validity assessment must continue to develop in order to meet the demands of changing demographics and healthcare factors (55). With respect to demographics, recent studies highlighted the necessity of adapting PVTs to demographic changing, thus increasing their applicability among international samples by establishing their accuracy and cross-validation in non-English-speaking populations (56). Furthermore, with respect to healthcare factors, the emergence of Information and Communication Technology and Artificial Intelligence (AI) could significantly improve our chance to detect and interpret malingering. AI could be considered, among other options. In fact, AI could significantly improve our chance to detect and interpret malingering, For instance, the use of wearable devices (in some cases a fitbank or, more simply, the mobile phone with specific apps) to detect signs of physical, cognitive and psychological impairment during everyday life (50, 57) might be helpful. Some special investigations technique, such as video surveillance, which is typically undertaken by the insurance companies, can provide useful information about the claimant's physical abilities. Marked or unexpected differences between the claimant's observed behaviors and what they claim to be unable to do, even if not definitive, can raise doubts as to the credibility of their report (8). However, new trends in research on malingering and technology (e.g., deception analysis by facial recognition) should also address ethical issues concerning individual privacy policies, especially in the realm of civil law (58). In addition, the possibility to administer specific tests to the subject/patient by electronic devices could strongly facilitate the unbiased detection of malingering/ identification of clinical impairment by the practitioner (57, 58). In this regard, AI, by the use of machine learning, could provide semi-automated diagnosis, under the control of an experienced physician. Making use of more advanced methodological (e.g., machine learning) would greatly enhance PVT's utility and applicability across a wider range of populations. It would also represent a fundamental shift from using idiosyncratic information about an individual examinee obtained from himself and/or significant others, to nomothetic data obtained by using vast databases and algorithms yielding descriptors and likely behaviors of the person of interest (59). Finally, additional research on validity testing *via* computer-based and telehealth modalities (60) also will be critical considering how the COVID-19 pandemic has resulted in opportunities for change in psychological/neuropsychological assessment practices.

## Data availability statement

The original contributions presented in the study are included in the article/supplementary material, further inquiries can be directed to the corresponding author.

## Ethics statement

Ethical review and approval was not required for the study on human participants in accordance with the local legislation and institutional requirements. The patients/participants provided their written informed consent to participate in this study. Written, informed consent was obtained from the participant for the publication of this case report (including all data).

## Author contributions

TB and FV observed the patient, collected and analyzed the data, and wrote the first draft of the manuscript. AV wrote the section Future directions and contributed to conceptualization and critical manuscript revision. RK substantially contributed to design malingering assessment and to literature review. All authors approved the final manuscript as submitted and agree to be accountable for all aspects of the work.

## Funding

This research was funded by the Ministry of Education, University and Research project Dipartimenti di Eccellenza 2018–2022 to Department of Neuroscience Rita Levi Montalcini.

## Conflict of interest

The authors declare that the research was conducted in the absence of any commercial or financial relationships that could be construed as a potential conflict of interest.

## Publisher's note

All claims expressed in this article are solely those of the authors and do not necessarily represent those of their affiliated organizations, or those of the publisher, the editors and the reviewers. Any product that may be evaluated in this article, or claim that may be made by its manufacturer, is not guaranteed or endorsed by the publisher.
